# Crystal structure of 5-amino-4*H*-1,2,4-triazol-1-ium pyrazine-2-carboxyl­ate: an unexpected salt arising from the deca­rboxylation of both precursors

**DOI:** 10.1107/S205698901501172X

**Published:** 2015-06-24

**Authors:** José A. Fernandes, Bing Liu, João P. C. Tomé, Luís Cunha-Silva, Filipe A. Almeida Paz

**Affiliations:** a Department of Chemistry, CICECO – Aveiro Institute of Materials, University of Aveiro, 3810-193 Aveiro, Portugal; b REQUIMTE / LAQV & Departamento de Química e Bioquímica, Faculdade de Ciencias, Universidade do Porto, 4169-007 Porto, Portugal; c Department of Chemistry, QOPNA, University of Aveiro, 3810-193 Aveiro, Portugal; d Department of Organic and Macromolecular Chemistry, Ghent University, B-9000 Ghent, Belgium

**Keywords:** crystal structure, ionothermal synthesis, deca­rboxylation, triazolium salt, hydrogen bonding, π–π stacking inter­actions

## Abstract

Both the 3-amino-2*H*,4*H*-1,2,4-triazolium cation and the pyrazine-2-carboxyl­ate anion in the title salt, C_2_H_5_N_4_^+^·C_5_H_3_N_2_O_2_^−^, were formed by an unexpected deca­rboxylation reaction, from 5-amino-1*H*-1,2,4-triazole-3-carb­oxy­lic acid and pyrazine-2,3-di­carb­oxy­lic acid, respectively. The dihedral angle between the pyrazine ring (r.m.s. deviation = 0.008 Å) and the carboxyl­ate group in the anion is 3.7 (3)°. The extended structure of the salt contains a supra­molecular zigzag tape in which cations and anions are engaged in strong and highly directional N—H⋯N,O hydrogen bonds, forming *R*_2_^2^(8) and *R*_2_^2^(9) graph-set motifs. The packing between the tapes is mediated by π–π stacking inter­actions between the triazole and pyrazine rings.

## Abstract 2

The salt 3-amino-2*H*,4*H*-1,2,4-triazolium pyrazine-2-carboxyl­ate was isolated by reacting 5-amino-1*H*-1,2,4-triazole-3-carb­oxy­lic acid and pyrazine-2,3-di­carb­oxy­lic acid in the presence of silver nitrate and 1-butyl-3-methyl­imidazolium bromide in ionothermal conditions.

## Chemical context   

A remarkable feature of ionothermal synthesis is the fact that ionic liquids (ILs) can act simultaneously as sustainable solvents and structure-directing agents (also known as templates). This has been widely demonstrated by their potential in the discovery of unprecedented crystalline mat­erials (Xu *et al.*, 2013 ▸). Following our inter­est in the design and preparation of new types of metal-organic frameworks (MOFs), we have been exploring the use of 5-amino-1*H*-1,2,4-triazole-3-carb­oxy­lic acid (H_2_atrc) and pyrazine-2,3-di­carb­oxy­lic acid (H_2_Pzdc) as a double-ligand system in the presence of transition metal centers using ionothermal synthetic conditions. In the presence of AgNO_3_ the obtained product revealed, however, to be an unexpected organic salt (Bond, 2007 ▸) composed of the 3-amino-2*H*,4*H*(+)-1,2,4-triazolium cation and the pyrazine-2-carboxyl­ato anion.
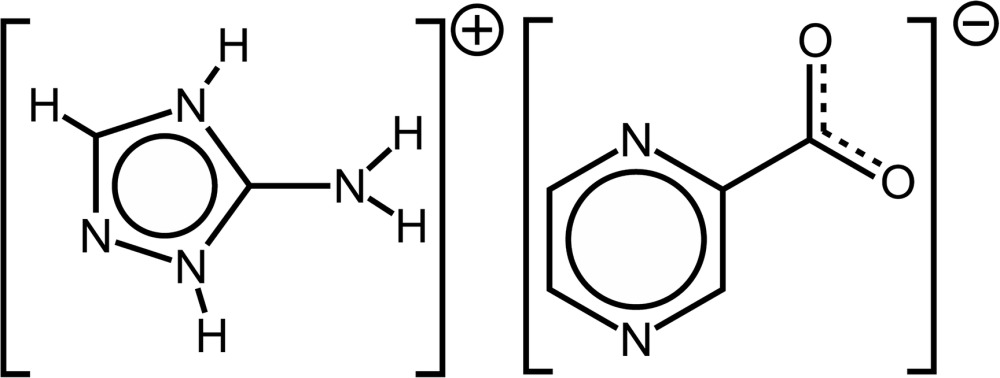


## Structural commentary   

**Figure 1:**
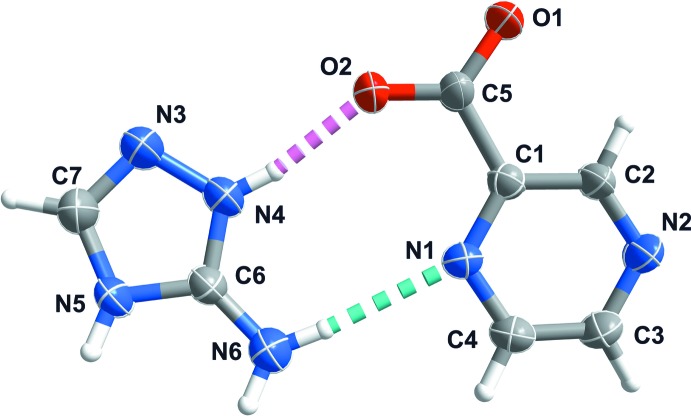
The asymmetric unit of the title salt. Non-H atoms are represented as displacement ellipsoids drawn at the 50% probability level, while H atoms are depicted as small spheres with arbitrary radii. The atomic labelling scheme for all non-H atoms is provided. Hydrogen bonds are represented as dashed lines.

The title compound is a product of decomposition of the H_2_atrc and H_2_Pzdc organic mol­ecules by way of decarb­oxyl­ation leading to, respectively, 3-amino-2*H*,4*H*-1,2,4-triazolium [(C_2_H_5_N_4_)^+^] and pyrazine-2-carboxyl­ate [(C_5_H_3_N_2_O_2_)^−^]. The asymmetric unit is composed of one of each of these moieties, as depicted in both the chemical diagram and in Fig. 1 ▸.

## Supra­molecular features   

**Figure 2:**
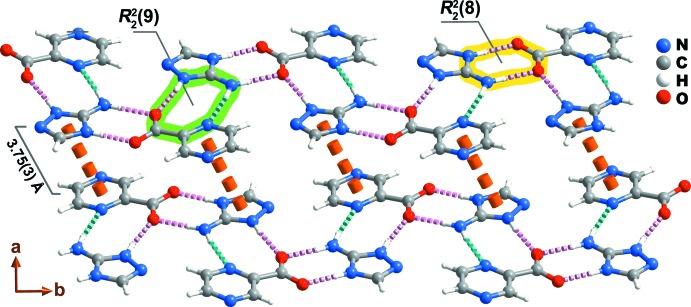
Supra­molecular tape running parallel to the [010] direction of the unit cell. N—H⋯N and N—H⋯N hydrogen bonds are depicted as dashed aqua and pink lines, respectively. Graph-set motifs present in the structure are highlighted. For geometric details of the represented supra­molecular contacts, see Table 1 ▸. π–π stacking inter­actions between two adjacent supra­molecular tapes are shown as orange dashed lines.

The cation present in the title compound is rich in groups capable of forming strong N—H⋯N,O hydrogen-bonding inter­actions (see Table 1 ▸ for further geometrical details), many highly directional with the observed <(*D*—H⋯*A*) inter­action angles being above 165°. These supra­molecular contacts are the main driving force which mediate the crystal packing features of the title compound. Indeed, the donation of hydrogen atoms from the cation to the carboxyl­ate group of an adjacent anion (N6—H6*B*⋯O2 and N5—H5⋯O1) forms the known structurally robust 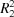
(8) graph-set motif (dashed pink lines in Fig. 2 ▸) (Grell *et al.*, 1999 ▸). This graph-set motif has already been found in salts containing the title compound cation and carb­oxy­lic acids (see *Database survey* below). Two other inter­actions, N6—H6*A*⋯N1 (dashed aqua lines) and N4—H4*A*⋯O2, describe a second 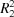
(9) hydrogen-bond motif. In contrast to the previous graph-set motif, the 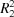
(9) ring has not been observed in structures containing the title-compound cation. The zigzag alternation of these two graph-set motifs leads to the formation of a highly coplanar supra­molecular tape running parallel to the [010] direction of the unit cell (Fig. 2 ▸). Adjacent tapes inter­act by way of weak π–π stacking contacts between triazole and pyrazine rings, with the inter-centroid distance being 3.75 (3) Å (dashed orange lines in Fig. 2 ▸).

## Database survey   

Triazole mol­ecules have been extensively used in the preparation of organic co-crystals (Kastelic *et al.*, 2011 ▸; Remenar *et al.*, 2003 ▸), and a survey of the Cambridge Structural Database (Groom & Allen, 2014 ▸) revealed the existence of about a dozen of crystallographic reports of co-crystals of the title compound cation (Byriel *et al.*, 1992 ▸; Essid *et al.*, 2013 ▸; Joo *et al.*, 2013 ▸; Luo *et al.*, 2013 ▸; Lynch *et al.*, 1992 ▸, 1998 ▸, 1999 ▸; Lynch, Smith, Byriel & Kennard, 1994 ▸; Lynch, Smith, Byriel, Kennard *et al.*, 1994 ▸; Matulková *et al.*, 2007 ▸; Smith *et al.*, 1996 ▸). The only compounds known with both of the title compound entities present is a bimetallic complex also containing Cd^2+^ and NO^3−^ ions (Chen *et al.*, 2009 ▸).

## Synthesis and crystallization   

5-Amino-1*H*-1,2,4-triazole-3-carb­oxy­lic acid (H_2_atrc, 98% purity), pyrazine-2,3-di­carb­oxy­lic acid (H_2_Pzdc, 97% purity), 1-methyl­imidazole (99%+ purity), 1-bromo­butane (99% purity) and AgNO_3_ (99%+ purity) were purchased from Sigma–Aldrich and were used as received without further purification. 1-Butyl-3-methyl­imidazolium bromide ([BMI]Br) was prepared according to the literature method (Parnham & Morris, 2006 ▸) and was isolated as a pale-yellow oil (yield of *ca* 78%).

AgNO_3_ (0.0687 g; 0.400 mmol), H_2_atrc (0.0510 g; 0.400 mmol) and H_2_Pzdc (0.0607 g; 0.361 mmol) were mixed with 0.49 g of [BMI]Br and 0.3 mL of distilled water in a *ca* 25 mL Teflon-lined stainless-steel reaction vessel. The resulting mixture was heated to 383 K for 7 days. The vessel was then allowed to cool to ambient temperature at a rate of *ca* 1 K h^−1^. Small colourless crystals of the title compound were directly isolated from the vessel contents.

## Refinement details   

Crystal data, data collection and structure refinement details are summarized in Table 2 ▸. Hydrogen atoms bound to carbon were placed at idealized positions with C—H = 0.95 Å, and included in the final structural model in a riding-motion approximation with the isotropic thermal displacement parameters fixed at 1.2*U*_eq_ of the carbon atom to which they are attached. Hydrogen atoms associated with nitro­gen atoms were located directly from difference Fourier maps and were included in the model with the N—H and H⋯H (only for the –NH_2_ groups) distances restrained to 0.90 (1) and 1.55 (1) Å, respectively, in order to ensure a chemically reasonable environment for these groups. These hydrogen atoms were modelled with the isotropic thermal displacement parameters fixed at 1.5*U*_eq_(N).

## Supplementary Material

Crystal structure: contains datablock(s) I, New_Global_Publ_Block. DOI: 10.1107/S205698901501172X/hb7446sup1.cif

Structure factors: contains datablock(s) I. DOI: 10.1107/S205698901501172X/hb7446Isup2.hkl

Supporting information file. DOI: 10.1107/S205698901501172X/hb7446Isup3.cml

CCDC reference: 1407396

Additional supporting information:  crystallographic information; 3D view; checkCIF report

## Figures and Tables

**Table 1:** Hydrogen-bond geometry (, )

*D*H*A*	*D*H	H*A*	*D* *A*	*D*H*A*
N4H4*A*O2	0.90(1)	1.77(1)	2.655(3)	166(3)
N5H5O1^i^	0.90(1)	1.73(1)	2.632(3)	176(3)
N6H6*B*O2^i^	0.90(1)	1.97(1)	2.853(3)	169(3)
N6H6*A*N1	0.90(1)	2.21(1)	3.099(3)	169(3)

Symmetry code: (i) 
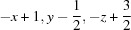
.

**Table 2:** Experimental details

Crystal data	
Chemical formula	C_2_H_5_N_4_^+^C_5_H_3_N_2_O_2_
*M*_r_	208.19
Crystal system, space group	Monoclinic, *P*2_1_/*c*
Temperature (K)	296
*a*, *b*, *c* ()	7.0599(5), 12.1868(8), 10.8385(6)
()	103.593(4)
*V* (^3^)	906.40(10)
*Z*	4
Radiation type	Mo *K*
(mm^1^)	0.12
Crystal size (mm)	0.09 0.04 0.03

Data collection	
Diffractometer	Bruker X8 Kappa CCD APEXII
Absorption correction	Multi-scan (*SADABS*; Sheldrick, 1998 ▸)
*T*_min_, *T*_max_	0.989, 0.997
No. of measured, independent and observed [*I* > 2(*I*)] reflections	12089, 1858, 1037
*R*_int_	0.077
(sin /)_max_ (^1^)	0.625

Refinement	
*R*[*F* ^2^ > 2(*F* ^2^)], *wR*(*F* ^2^), *S*	0.059, 0.133, 1.02
No. of reflections	1858
No. of parameters	148
No. of restraints	5
H-atom treatment	H atoms treated by a mixture of independent and constrained refinement
_max_, _min_ (e ^3^)	0.19, 0.20

Computer programs: *APEX2* (Bruker, 2006 ▸), *SAINT-Plus* (Bruker, 2005 ▸), *SHELXS97* and *SHELXTL* (Sheldrick, 2008 ▸), *SHELXL2014* (Sheldrick, 2015 ▸) and *DIAMOND* (Brandenburg, 2009 ▸).

## supplementary crystallographic information

### Crystal data

**Table:**

C_2_H_5_N_4_^+^·C_5_H_3_N_2_O_2_^−^	*F*(000) = 432
*M**_r_* = 208.19	*D*_x_ = 1.526 Mg m^−^^3^
Monoclinic, *P*2_1_/*c*	Mo *K*α radiation, λ = 0.71073 Å
*a* = 7.0599 (5) Å	Cell parameters from 1298 reflections
*b* = 12.1868 (8) Å	θ = 2.6–19.7°
*c* = 10.8385 (6) Å	µ = 0.12 mm^−^^1^
β = 103.593 (4)°	*T* = 296 K
*V* = 906.40 (10) Å^3^	Block, colourless
*Z* = 4	0.09 × 0.04 × 0.03 mm

### Data collection

**Table:**

Bruker X8 Kappa CCD APEXII diffractometer	1037 reflections with *I* > 2σ(*I*)
Radiation source: fine-focus sealed tube	*R*_int_ = 0.077
ω / φ scans	θ_max_ = 26.4°, θ_min_ = 2.6°
Absorption correction: multi-scan (*SADABS*; Sheldrick, 1998)	*h* = −8→8
*T*_min_ = 0.989, *T*_max_ = 0.997	*k* = −15→15
12089 measured reflections	*l* = −13→13
1858 independent reflections	

### Refinement

**Table:**

Refinement on *F*^2^	5 restraints
Least-squares matrix: full	Hydrogen site location: mixed
*R*[*F*^2^ > 2σ(*F*^2^)] = 0.059	H atoms treated by a mixture of independent and constrained refinement
*wR*(*F*^2^) = 0.133	*w* = 1/[σ^2^(*F*_o_^2^) + (0.0599*P*)^2^] where *P* = (*F*_o_^2^ + 2*F*_c_^2^)/3
*S* = 1.01	(Δ/σ)_max_ < 0.001
1858 reflections	Δρ_max_ = 0.19 e Å^−^^3^
148 parameters	Δρ_min_ = −0.20 e Å^−^^3^

### Special details

**Table:**

Geometry. All e.s.d.'s (except the e.s.d. in the dihedral angle between two l.s. planes) are estimated using the full covariance matrix. The cell e.s.d.'s are taken into account individually in the estimation of e.s.d.'s in distances, angles and torsion angles; correlations between e.s.d.'s in cell parameters are only used when they are defined by crystal symmetry. An approximate (isotropic) treatment of cell e.s.d.'s is used for estimating e.s.d.'s involving l.s. planes.

### Fractional atomic coordinates and isotropic or equivalent isotropic displacement parameters (Å^2^)

**Table:**

	*x*	*y*	*z*	*U*_iso_*/*U*_eq_	
N1	0.2890 (3)	0.79063 (17)	0.49872 (19)	0.0389 (6)	
N2	0.0664 (3)	0.79145 (18)	0.2472 (2)	0.0468 (6)	
C1	0.2177 (4)	0.8840 (2)	0.4404 (2)	0.0334 (6)	
C2	0.1066 (4)	0.8815 (2)	0.3165 (2)	0.0412 (7)	
H2	0.0571	0.9476	0.2797	0.049*	
C3	0.1381 (4)	0.6995 (2)	0.3059 (2)	0.0448 (7)	
H3	0.1147	0.6334	0.2623	0.054*	
C4	0.2461 (4)	0.6993 (2)	0.4297 (3)	0.0442 (7)	
H4	0.2912	0.6326	0.4668	0.053*	
C5	0.2560 (4)	0.9921 (2)	0.5101 (2)	0.0378 (7)	
O1	0.1745 (3)	1.07442 (14)	0.45075 (15)	0.0481 (6)	
O2	0.3628 (3)	0.99415 (14)	0.61986 (16)	0.0543 (6)	
N3	0.7128 (4)	0.94320 (19)	0.8957 (2)	0.0622 (8)	
N4	0.6167 (4)	0.88117 (19)	0.7929 (2)	0.0476 (6)	
H4A	0.535 (3)	0.912 (2)	0.7253 (18)	0.071*	
N5	0.7480 (3)	0.76575 (18)	0.93596 (19)	0.0417 (6)	
H5	0.778 (4)	0.6996 (13)	0.972 (2)	0.063*	
N6	0.5628 (4)	0.6942 (2)	0.7405 (2)	0.0540 (7)	
H6A	0.473 (3)	0.714 (2)	0.6709 (19)	0.081*	
H6B	0.585 (5)	0.6271 (13)	0.775 (3)	0.081*	
C6	0.6369 (4)	0.7751 (2)	0.8181 (2)	0.0365 (7)	
C7	0.7892 (5)	0.8695 (2)	0.9782 (3)	0.0553 (8)	
H7	0.8649	0.8861	1.0584	0.066*	

### Atomic displacement parameters (Å^2^)

**Table:**

	*U*^11^	*U*^22^	*U*^33^	*U*^12^	*U*^13^	*U*^23^
N1	0.0450 (14)	0.0290 (13)	0.0400 (12)	−0.0006 (11)	0.0049 (10)	0.0005 (10)
N2	0.0557 (16)	0.0387 (14)	0.0406 (12)	0.0008 (12)	0.0005 (11)	−0.0086 (12)
C1	0.0331 (15)	0.0309 (15)	0.0355 (13)	−0.0004 (13)	0.0067 (11)	0.0022 (12)
C2	0.0495 (18)	0.0320 (16)	0.0367 (14)	0.0061 (14)	−0.0007 (13)	0.0020 (12)
C3	0.0487 (19)	0.0352 (17)	0.0478 (16)	−0.0016 (15)	0.0059 (14)	−0.0099 (13)
C4	0.0499 (18)	0.0299 (16)	0.0497 (16)	0.0036 (14)	0.0056 (14)	0.0028 (13)
C5	0.0420 (17)	0.0344 (16)	0.0330 (13)	0.0001 (14)	0.0006 (12)	0.0036 (12)
O1	0.0625 (14)	0.0319 (11)	0.0402 (10)	0.0058 (9)	−0.0074 (9)	0.0021 (8)
O2	0.0708 (14)	0.0375 (12)	0.0399 (10)	0.0067 (10)	−0.0165 (10)	−0.0025 (9)
N3	0.089 (2)	0.0419 (15)	0.0461 (14)	0.0029 (15)	−0.0032 (13)	0.0024 (12)
N4	0.0625 (18)	0.0367 (15)	0.0386 (13)	0.0037 (13)	0.0019 (12)	0.0061 (11)
N5	0.0480 (14)	0.0362 (15)	0.0359 (12)	0.0025 (12)	−0.0004 (11)	0.0083 (11)
N6	0.0607 (18)	0.0440 (16)	0.0491 (15)	−0.0005 (15)	−0.0038 (13)	0.0030 (13)
C6	0.0391 (17)	0.0349 (18)	0.0348 (13)	0.0044 (13)	0.0068 (12)	0.0057 (12)
C7	0.074 (2)	0.046 (2)	0.0383 (15)	0.0020 (17)	−0.0026 (15)	0.0012 (14)

### Geometric parameters (Å, º)

**Table:**

N1—C4	1.335 (3)	N3—C7	1.293 (3)
N1—C1	1.341 (3)	N3—N4	1.384 (3)
N2—C2	1.323 (3)	N4—C6	1.322 (3)
N2—C3	1.328 (3)	N4—H4A	0.902 (10)
C1—C2	1.387 (3)	N5—C6	1.338 (3)
C1—C5	1.512 (3)	N5—C7	1.353 (3)
C2—H2	0.9300	N5—H5	0.901 (10)
C3—C4	1.379 (4)	N6—C6	1.321 (3)
C3—H3	0.9300	N6—H6A	0.899 (10)
C4—H4	0.9300	N6—H6B	0.896 (10)
C5—O2	1.250 (2)	C7—H7	0.9300
C5—O1	1.256 (3)		
			
C4—N1—C1	115.6 (2)	C7—N3—N4	102.9 (2)
C2—N2—C3	114.9 (2)	C6—N4—N3	111.1 (2)
N1—C1—C2	120.2 (2)	C6—N4—H4A	126 (2)
N1—C1—C5	120.1 (2)	N3—N4—H4A	121.7 (19)
C2—C1—C5	119.8 (2)	C6—N5—C7	105.9 (2)
N2—C2—C1	124.4 (2)	C6—N5—H5	121.3 (18)
N2—C2—H2	117.8	C7—N5—H5	132.7 (18)
C1—C2—H2	117.8	C6—N6—H6A	115 (2)
N2—C3—C4	122.0 (2)	C6—N6—H6B	115 (2)
N2—C3—H3	119.0	H6A—N6—H6B	128 (3)
C4—C3—H3	119.0	N6—C6—N4	126.2 (2)
N1—C4—C3	123.0 (2)	N6—C6—N5	126.9 (2)
N1—C4—H4	118.5	N4—C6—N5	106.9 (2)
C3—C4—H4	118.5	N3—C7—N5	113.2 (2)
O2—C5—O1	125.0 (2)	N3—C7—H7	123.4
O2—C5—C1	119.3 (2)	N5—C7—H7	123.4
O1—C5—C1	115.7 (2)		
			
C4—N1—C1—C2	0.3 (4)	N1—C1—C5—O1	−176.1 (2)
C4—N1—C1—C5	179.5 (2)	C2—C1—C5—O1	3.1 (4)
C3—N2—C2—C1	1.2 (4)	C7—N3—N4—C6	0.8 (3)
N1—C1—C2—N2	−1.4 (4)	N3—N4—C6—N6	179.8 (3)
C5—C1—C2—N2	179.4 (2)	N3—N4—C6—N5	−0.9 (3)
C2—N2—C3—C4	−0.1 (4)	C7—N5—C6—N6	179.9 (3)
C1—N1—C4—C3	0.8 (4)	C7—N5—C6—N4	0.6 (3)
N2—C3—C4—N1	−1.0 (4)	N4—N3—C7—N5	−0.4 (4)
N1—C1—C5—O2	3.7 (4)	C6—N5—C7—N3	−0.1 (4)
C2—C1—C5—O2	−177.1 (2)		

### Hydrogen-bond geometry (Å, º)

**Table:**

*D*—H···*A*	*D*—H	H···*A*	*D*···*A*	*D*—H···*A*
N4—H4*A*···O2	0.90 (1)	1.77 (1)	2.655 (3)	166 (3)
N5—H5···O1^i^	0.90 (1)	1.73 (1)	2.632 (3)	176 (3)
N6—H6*B*···O2^i^	0.90 (1)	1.97 (1)	2.853 (3)	169 (3)
N6—H6*A*···N1	0.90 (1)	2.21 (1)	3.099 (3)	169 (3)

Symmetry code: (i) −*x*+1, *y*−1/2, −*z*+3/2.
